# Real-time polarization compensation method in quantum communication based on channel Muller parameters detection

**DOI:** 10.1038/s44172-024-00198-0

**Published:** 2024-03-22

**Authors:** Yongjian Tan, Jianyu Wang, Jincai Wu, Zhiping He

**Affiliations:** 1 Key Laboratory of Space Active Optoelectronic Technology, Shanghai Institute of Technical Physics, Chinese Academy of Sciences, Shanghai, 200083 China; 2 University of Chinese Academy of Sciences, Beijing, 100049 China; 3 Shanghai Quantum Science Research Center, Shanghai, 201315 China; 4 Hefei National Laboratory, Hefei, 230088 China

**Keywords:** Fibre optics and optical communications, Optomechanics, Quantum information

## Abstract

Polarization drift in fiber and free-space optical links is a major factor in the dynamic increase of bit error rate in polarization-coded quantum key distribution (QKD) systems. A dynamic polarization compensation method applicable to both links is a challenge. Here we propose a universally applicable real-time polarization compensation method, that the Muller parameters of the optical links are first detected using a polarization detector, and then the optimal parameters of the controller are obtained by gradient descent algorithm. Simulation results indicate advantages over current methods, with fewer waveplates, faster speed, and wider applicability for various optical links. In equivalent experiments of both satellite and fiber optical links, the average polarization extinction ratio of 27.9 dB and 32.2 dB are respectively achieved. The successful implementation of our method will contribute to the real-time polarization design of fiber and free-space QKD systems, while also contributing to the design of laser-based polarization systems.

## Abstract (web summary)

Yongjian Tan and colleagues report a universally applicable real-time polarization compensation method for quantum communications. The approach has several advantages over current methods, including a minimum number of waveplates, faster speed, and wider applicability for various optical links.

## Introduction

Quantum key distribution is the intrinsically unconditional secure method of transmitting keys based on the principles of quantum mechanics^1,2^. It is currently the most mature and commonly employed technology in quantum communication. The distribution of keys through encoded photons, either via fiber or free-space optical link, is the predominant method for implementing quantum key distribution^3^. The construction of an integrated global QKD network based on these two optical links is becoming the prevailing trend in quantum communication^4–6^.

There are three primary coding methods employed in QKD^3^: time bin, phase, and polarization. At present, polarization coding is the prevailing method utilized in both fiber and free-space. High-fidelity transmission of encoded photons is essential for successful key distribution. Besides the inherent polarization degradation induced by optical devices^7,8^, environmental perturbations, variations in telescope attitude^9^, and jitter in basis vectors^10^ between the transmitter and receiver can readily lead to dynamic polarization degradation. In the case of optical fiber link^11^ even slight disturbances can result in substantial alterations to the polarization state. Similarly, in the case of free-space optical link, the light source is typically coupled into free space using optical fiber^12^. Although the dynamic degradation of optical fibers may be relatively modest, when coupled with the continuously changing telescope attitude and basis vector jitter, it leads to substantial dynamic polarization degradation. All of the above will cause the QBER to rise sharply. Consequently, real-time compensation of the polarization of the optical link is imperative to achieve high-fidelity transmission of polarization-encoded quantum states.

To sustain the polarization performance of the optical link, polarization-maintaining fibers and mirror coating techniques^11^ are frequently employed. However, these methods are ineffective in handling dynamic changes^9^ in the polarization state and are expensive and difficult to implement on a large scale. The polarization controller-based real-time polarization compensation technology has played a critical role in both fiber and free-space optical links^13^. This technology maintains the polarization state at high quality with only a few waveplates.

Real-time compensation of polarization states typically involves searching for optimal parameters(such as voltage, rotation angle, temperature, etc. that can change the position or phase delay of the fast/slow axes of the wave plate) of polarization controller, including piezo electric polarization controller (EPC)^14^, liquid-crystal variable retarders (LCVR)^15^, lithium niobite (LiNbO3) crystal^16^, and crystal waveplate group(WPG)^11^, among others, using a polarization detection system. To identify these optimal parameters, feedback control algorithms, including stochastic parallel gradient descent (SPGD) algorithm^17^, Jacobian algorithm^18^, and fast-locating algorithm^19^, are commonly employed. These algorithms adjust the parameters of controller continuously to achieve an iterative approximation of the lowest quantum bit error rate (QBER). However, this approach takes some time to converge and requires a considerable amount of detection resources, making it unsuitable for high-frequency optical links, such as buried fibers^20^ and aerial fibers^21^. To shorten the iteration time, a heuristic polarization compensation method has been proposed^22^, which reduces the iteration time by detecting the QBER in up to six steps. Heuristic method is of paramount significance in the real-time compensation of fiber optical link. Nonetheless, its applicability to a broader spectrum of scenarios is limited due to its omission of polarization degradation occurring between the polarization controller and the detector. As an illustrative example, in free-space optical link, encoded light is commonly coupled to free space via optical fiber, frequently with a polarization controller located at the transmitter^11,12,23–25^. In another example, the detector used is the fiber-coupled superconducting nanowire single photon detector (SNSPD), and the polarization degradation of the coupled fiber is difficult to disregard^26^. The waveplate group polarization compensation method has been successfully applied to the free-space optical link^27^, and real-time dynamic polarization control of the transmitter optical path has been achieved using the waveplate group through the analytical method^9^. However, this method requires measuring the parameters of each optical component, and additional detection instruments increase measurement costs. Although these parameters can be measured in advance to reduce the number of detectors, it is limited to optical links with slow polarization parameter changes.

The methods described above have been valuable within their specific scenarios; nevertheless, a universally applicable, fast, real-time polarization control method remains elusive. The polarization transmission process of any QKD optical link, whether it involves fiber optics or free space, can be characterized by a polarization transmission matrix, which is because quantum communication requires the transmission matrix of the channel to obey unitary transformation^2^. It is noteworthy that the three-waveplate method can compensate for polarization degradation in systems that obey unitary transformation^28^. This method has found application in polarization compensation for fiber^28^, free-space^27^, and hybrid optical paths^9^, affirming the existence of the universal polarization transmission model. The core objective of polarization control is to transform the transmission matrix of an optical link into a unit matrix. Analyzing the polarization transmission matrix of the entire optical link allows for the determination of a universal polarization control method and its optimal configuration. As far as our knowledge extends, this analysis has not been undertaken by anyone.

In this paper, we introduce a real-time polarization control method based on a universal polarization transmission model that represented by the Muller matrix. The method utilizes the polarization detector situated at the optical link’s terminus to detect the channel Muller parameters based on the adjustable characteristics of the transmission matrix of the polarization controller. After completing the parameter detection, the optimal transmission matrix of the polarization controller is solved. By converting the parameter detection model into a system of linear equations, the minimum number of required detections in different scenarios can be determined, reducing the method’s time consumption. Simulation results for fiber optical link and satellite-ground optical link indicate that this method has several advantages over current methods, including requiring a minimum number of waveplates, faster speed, and wider applicability for various optical links. Moreover, it shows potential for real-time polarization control over full optical link of satellite-ground QKD. We experimentally verified the effectiveness of this method using a crystal waveplate group and achieved a high polarization extinction ratio (PER) in polarization control. Successfully implementing this method will facilitate the design of efficient and low-cost real-time polarization control for fiber and free-space QKD and play an essential role in polarization design for other laser-based systems.

## Results and discussion

### Simulation verification

In this section, we will apply our method to two types of optical links. The first type is a fiber-based optical link where the polarization controller is typically placed near the detector at the receiver (*M*_2_ = *E*). We will simulate and compare the SPGD method, heuristic method. The second type is a satellite-ground optical link, where the polarization controller is typically located in the transmitter, and both *M*_1_ and *M*_2_ are not identity matrices. In this case, the heuristic method will fail, which requires that *M*_2_ must be the identity matrix. We will evaluate the effectiveness of our method by applying it to two schemes: satellite optical link compensation and full optical link compensation. The former exclusively addresses compensation for the optical path within the satellite transmitter. This scheme is currently prevalent because of the minor polarization degradation in the ground receiver, which can be attributed to the use of the free-space decoding module. In contrast, the latter scheme involves compensation for the entire optical link, spanning from the light source within the transmitter to the ground receiver.

#### Fiber optical link

The random polarization drift will be generated in fiber optical link, which can be represented by the Mueller matrix using the model described in ref. ^29^:

1 $${M}_{t}=M\left({{{{\boldsymbol{\kappa }}}}}_{{{{\boldsymbol{t}}}}}\right){M}_{t-1}$$

*M*_*t* − 1_ represents the Mueller matrix at the previous time step, and M_*t*_ represents the current Mueller matrix. *M*(*κ*_*t*_) is defined as follows:

2 $$M\left({{{\boldsymbol{\kappa }}}}\right)={{{{\rm{I}}}}}_{3}+\sin (2\alpha )\chi ({{{\bf{a}}}})+(1-\cos (2\alpha ))\chi {({{{\bf{a}}}})}^{2}$$

I_3_ is a 3 × 3 identity matrix. *χ*(**a**) is represented as:

3 $$\chi ({{{\bf{a}}}})=\left(\begin{array}{ccc}0 & -{a}_{3} & {a}_{2}\\ {a}_{3} & 0 & -{a}_{1}\\ -{a}_{2} & {a}_{1} & 0\end{array}\right)$$

Where ***κ*** = *α* ***a***, ***a*** = (*a*_1_, *a*_2_, *a*_3_), *α* = ||***κ***||. ||·|| represents the Euclidean norm. ***κ***_***t***_ follows a zero-mean normal distribution at time t, i.e., ${{{{\boldsymbol{\kappa }}}}}_{{{{\boldsymbol{t}}}}}{\sim }{{{\mathscr{N}}}}({{{\bf{0}}}},{\sigma }_{p}^{2}{{{{\rm{I}}}}}_{3})$. ${\sigma }_{p}^{2}=2\pi \varDelta {pT}$, where *T* is the time interval between *M*_*t*−1_ and *M*_*t*_, and *Δp* is the polarization linewidth which determines the speed of polarization drift.

**Fig. 1:**
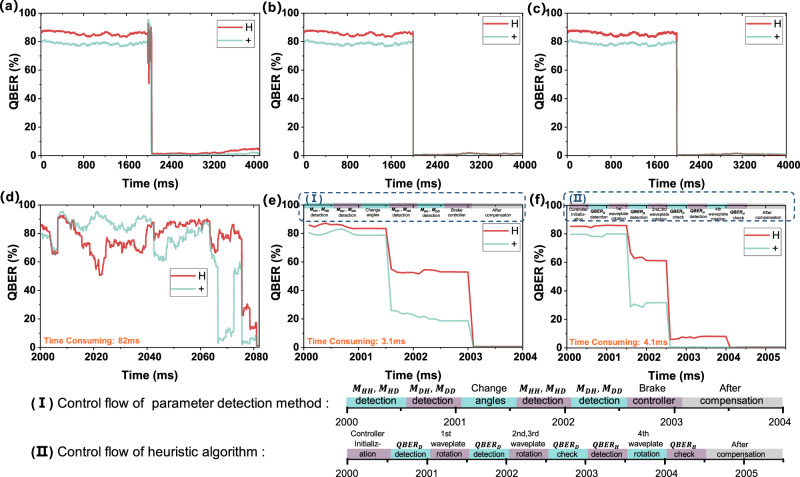
Comparison of three compensation methods for fiber optical link. The simulated QBER timing diagram of (**a**) SPGD method, (**b**) channel Muller parameters detection method, and (**c**) heuristic method; The QBER timing diagram during the control stage of (**d**) SPGD method, (**e**) channel Muller parameters detection, and (**f**) heuristic method.

In the simulated optical link model, it is assumed that the impact of channel loss is ignored and only polarization effects are considered. The reference for this study is the 16.9 km WTPT-KLQI fiber optical link^20^, which experienced a 1% increase in average QBER within 1 minute. Simulation calculations indicate that *Δp* should be set to 4 × 10^−5^ Hz. The source’s degree of polarization is 0.995, while the single photon detector acquisition time is 0.5 ms, and EPC polarization controller has a braking time of 0.5 ms as well. The “interruptions” scheme^30^ (the polarization parameters of the optical link are obtained by extracting the polarization characteristics of the quantum light used for key distribution, thus the key distribution will be interrupted during the polarization control process) is adopted to detect the polarization parameters, where both the polarization detection and decoding module share the same system. The fiber matrix is initially set as a random quantity. After 2000 ms of random drift, polarization control is executed. The additional 2000 ms of polarization performance are observed after the completion of the control. The QBER for H and + directions are shown in Fig. 1.

Both Muller parameters detection method and heuristic method can achieve almost perfect polarization control in a short time. However, the SPGD method’s execution time heavily relies on the initial parameters, making the process longer than the other two methods. Table 1 provides a summary of the comparison among these three methods.

**Table 1:** Compensation methods comparison in fiber optical link

Type	Controller type	Speed	Number of components	Compensable optical link location
Heuristic	Delay adjustable and Fast/Slow axes rotatable	Fast	4	Between controller and detector
Feedback control	All type	Slow	2, 3	Full optical link
parameter detection	All types with known parameters	Fast	2, 3	Full optical link

#### Satellite-ground optical link

**Fig. 2:**
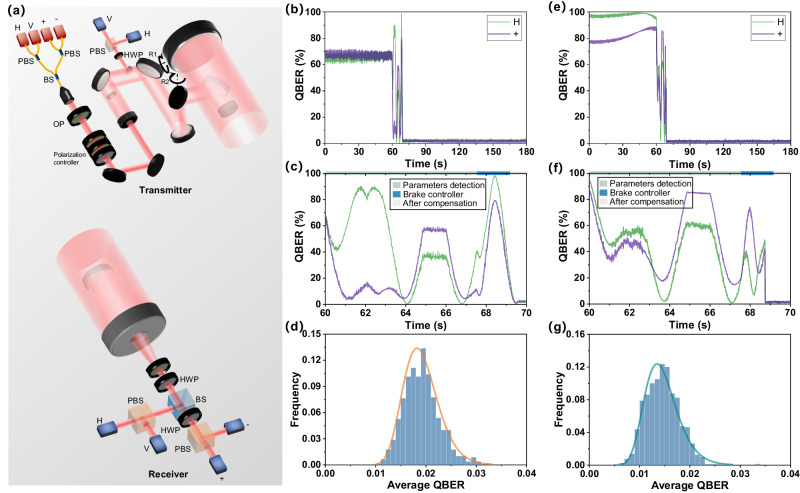
Polarization compensation in the satellite-ground optical link. **a** Setup of the receiver and transmitter; simulated QBER timing diagram of channel Muller parameters method (**b**) applied to satellite optical link, and (**e**) that combines with dynamic compensation scheme; simulated QBER timing diagram in control stage of channel Muller parameters method (**c**) applied to satellite optical link, and (**f**) that combines with dynamic compensation scheme; simulated average QBER frequency distribution after compensation of channel Muller parameters method (**d**) applied to satellite optical link, and (**g**) that combines with dynamic compensation scheme. PBS polarization beam splitter, BS beam splitter, OP front-end optical path, HWP 1/2 waveplate.

In the satellite-ground optical link, a complex optical link configuration is considered as shown in Fig. 2a. The encoding quantum light successively passes through fiber, front-end optical path, polarization controller, back-end optical path, telescope system, and then reaches the ground receiver. The ground receiver is equipped with a 1/2 waveplate to compensate for the basic vector deviation. However, due to the phase delay of the mirrors, even if the basis vectors are corrected, rotation of azimuth and elevation axes will still lead to dynamic changes in the polarization state. The satellite telescope system adopts the Coude type^31^, whose rotation of the azimuth axis and elevation axis will cause the deviation of basis vectors. The ground telescope adopts the Cassegrain type^31^ which does not affect basic vectors during rotation.

The polarization controller is a waveplate group composed of crystal waveplates, which are driven by the motor to rotate the fast and slow axes. We assume a channel loss of 40 dB and a light source repetition frequency of 1 GHz. Consequently, the average photon count at the detector per second is 10^5^, with an average time interval of 10 μs. This interval exceeds the typical dead time of common single-photon detectors, approximately 100 ns. To simplify our calculations, we assume that these photons arrive at the detector evenly at 10μs intervals.

The optical link polarization transmission model is represented as follows:

4 $${M}_{P}={M}_{H}{M}_{\delta 4}{M}_{Ts}{M}_{\delta 3}{M}_{T3}{M}_{\delta 2}{M}_{T2}{M}_{\delta 1}{M}_{c}{M}_{\delta 0}{M}_{F}$$

*M*_*F*_ is the transmission matrix of the fiber, and *M*_*H*_ is the transmission matrix of the 1/2 waveplate at the receiver. The simulation parameters are shown in the Table 2:

**Table 2:** Simulation parameters of satellite-ground link

Parameters	Symbol	Value
Polarization linewidth	*Δp*	40 nHz
Azimuth, elevation and relative rotation angle of the telescope tubes	*T*2, *T*3, *Ts*	Simulation data of STK in ref. ^32^
Satellite telescope delay	*δ*2,*δ*3	6°, 6°
Ground telescope delay	*δ*4	6°
Front-end optical system delay	*δ*0	20°
Back-end optical system delay	*δ*1	20°
Basis vector compensation error	-	${{{\mathscr{N}}}}{\sim }\left(0,{0.2}^{\circ }\right)$
Ground-to-satellite control signal transmission time	-	0.3 s
Waveplate rotation speed	-	40°/s
Detection time	-	10 ms
Degree of polarization	-	0.995
Channel loss	-	40 dB
Light source repetition frequency		1 GHz
Dark count rate		200 Hz

The telescope attitude data (120 s ~ 300 s) in a low-orbit satellite-to-ground optical link simulated by STK^32^ will be used to analyze the effect of our method applied to two polarization control schemes: satellite optical link control and full optical link control.

Polarization control in the satellite optical link is a commonly adopted approach to achieve polarization state control of the satellite-ground optical link. In our configuration, the polarization detector is installed in the satellite optical link to detect and monitor the polarization information through the cut beam. A 1/2 waveplate is utilized in the polarization detector to toggle between H/V and +/− channels, enabling the detection of channel Muller parameters. In this case *M*_1_ = *M*_*δ*0_ *M*_*F*_, *M*_2_ = *M*_*δ*1_ in Eq. (5). The simulation results are shown in Fig. 2b–d.

The application of this method in the satellite optical link control scheme has demonstrated its ability to achieve good control results. The polarization state controlling process in the satellite-ground optical link can be executed within 10 s, primarily dependent on the waveplate’s rotation speed. This duration is shorter than the duration of satellite passes, making real-time polarization control achievable with this method. The QBER is less than 3% with a 99% probability and less than 2% with a 63% probability. This is mainly due to the good polarization-maintaining design of the transmitter telescope and the receiver optical path (each part has only a 6° phase delay), as well as the low basis vector compensation error.

In this scheme, it is impossible to compensate for the polarization delay induced by the telescope positioned behind the detector. Although applying polarization-maintaining film can mitigate the influence of the telescope delay on the overall polarization-maintaining, this approach increases both technical complexity and cost. The dynamic polarization compensation scheme does not necessitate a flawless coating design; instead, it relies on advance acquisition of telescope delay and rotation attitude to attain a comparatively flawless compensation for the entire satellite optical link. The control method based on the Mueller parameter detection we introduced can detect the Mueller matrix (*M*_*δ*1_, *M*_*δ*0_ *M*_*F*_) of the optical link positioned in front of the detector, which can be complementary to the dynamic compensation scheme. At this time, in Eq. (5), *M*_1_ = *M*_*δ*0_ *M*_*F*_, *M*_2_ = *M*_*δ*3_ *M*_*T*3_ *M*_*δ*2_ *M*_*T*2_ *M*_*δ*1_. Since *M*_*T*2_, *M*_*T*3_, *M*_*δ*2_, and *M*_*δ*3_ are known, thus the parameters of the polarization controller can be obtained through Eq. (16). We applied the dynamic compensation scheme to our method and performed simulation verification.

After solving the parameters of each section optical link located in front of the detector, we can implement compensation for the entire satellite optical system by combining the known attitude and delay parameters of the telescope. The basis vector deviation between the telescope tubes will be corrected by using the 1/2 waveplate on the ground. The simulation results are as shown in Fig. 2e–g:

Due to the uncompensated delay of the telescope system, its effect is still inferior to its integration with dynamic compensation. The latter has a higher probability of achieving a QBER lower than 2% at approximately 94.5%. It is noteworthy that, with the polarization-maintaining coating design, the control method introduced in this paper can easily attain the same effect as its integration with dynamic compensation. Nevertheless, the scheme that integrates with dynamic compensation requires prior knowledge of the telescope delay, which limits its practicality when the telescope delay degrades.

Controlling only the transmitter optical link is currently the most widely used scheme. However, this necessitates ensuring that the receiver’s optical path is equipped with an effective polarization-maintaining design. We next apply our method to the entire optical link control. In this application, the polarization design of the whole link system and separate polarization detection of each component is unnecessary, which reduces costs and simplifies the polarization design. Nevertheless, due to the intricate dynamic modulation effect on the polarization state, it is challenging to implement corresponding control.

**Fig. 3:**
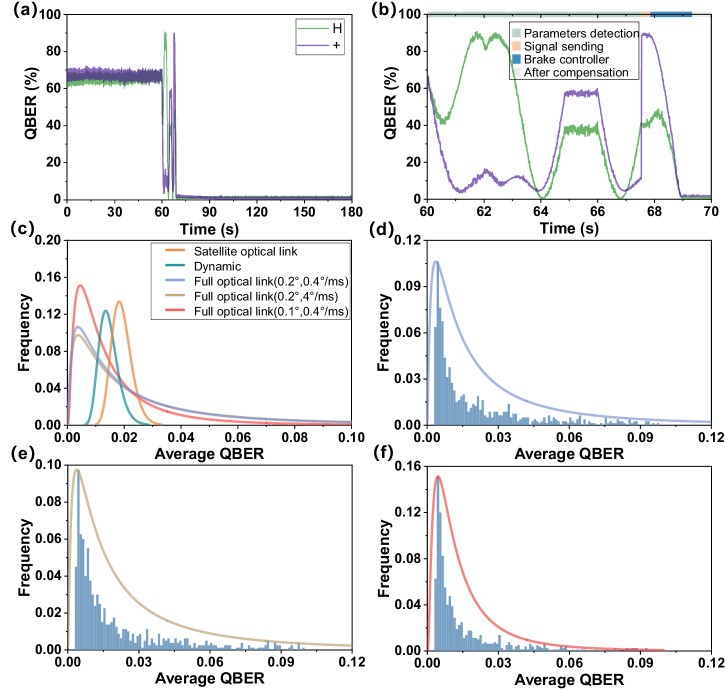
Simulated compensation results for the full optical link compensation scheme in satellite-ground optical link. **a** QBER timing diagram for the full optical link compensation scheme; (**b**) QBER timing diagram in control stage for the full optical link compensation scheme; (**c**) average QBER frequency distribution curve (log-normal distribution fitting^34^, this fitting method was chosen because it is closest to the histogram contour) after compensation; average QBER frequency distribution after compensation for (**d**) the full optical link compensation scheme with Table 2 parameters, (**e**) the waveplate rotation speed at 4°/ms, and (**f**) the basis vector compensation error at 0.1°.

As shown in Fig. 2a, in the full optical link compensation scheme, the polarization detector is located at the receiver and shares the same system with the BB84 decoding module. The polarization detector will collect the channel polarization signal modulated by in-orbit waveplates. Once the waveplate angles have been solved, they are transmitted to the satellite to enable polarization control. At this time, in Equation (5), *M*_1_ = *M*_*δ*0_ *M*_*F*_, *M*_2_ = *M*_*H*_ *M*_*δ*4_ *M*_*Ts*_ *M*_*δ*3_ *M*_*T*3_ *M*_*δ*2_ *M*_*T*2_ *M*_*δ*1_. The compensation results are shown in Fig. 3.

In the full optical link compensation scheme, achieving a lower QBER is easier than in the satellite optical link compensation. However, the full optical link compensation scheme has poor repeatability, with only a 70% probability that the QBER is less than 3%. This is mainly due to additional detection errors introduced by compensation errors in the basis vectors, dynamic changes in system polarization, and the slow rotation of the waveplate. Nevertheless, it is noteworthy that our method still shows the potential to be applied for full optical link compensation, as shown in Fig. 3c. In particular, when the compensation error in the basis vectors is reduced to 0.1°, the repeatability is improved, with an 84% probability that the QBER is less than 3%.

In short, the compensation effect of our method is limited by the jitter of channel Muller parameters. The method is very suitable for optical links with Muller parameters that are quasi-statically within the control time, such as fiber and satellite optical systems. The compensation effect and robustness will improve once the Muller parameters jitter reduces.

### Experiments verification

**Fig. 4:**
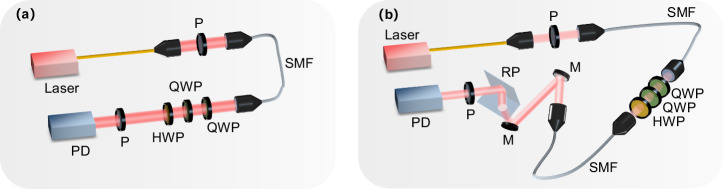
Schematic diagram of experimental setup. **a** Fiber optical link; (**b**) simulated satellite optical link. P polarizer, SMF single-mode fiber, QWP 1/4 waveplate, HWP 1/2 waveplate, M mirror, RP roof prism, PD power detector.

To verify the effectiveness of the compensation method, two equivalent experiments for quasistatic optical links were implemented: one is the control for the fiber optical link and the other is for the simulated satellite optical link, as shown in Fig. 4. The beam emitted from the laser source (Shanghai Fiblaser, 850 nm Fiber Coupled Laser) is encoded by a polarizer and coupled into a 1 m fiber (Thorlabs,780HP) through a fiber coupler, then reaches the quartz waveplate group composed of two QWPs (LBTEK, QWP20-850B), and HWP (LBTEK, HWP20-850B). In the fiber link, after passing through the waveplate group, the beam will reach the detection system consisting of a linearly polarizer and power meter to detect the channel Muller parameters and compensate for the polarization degradation. In the simulated satellite optical link, the backend optical path is composed of fibers, two aluminum-coated mirrors (Thorlabs, PFE10-G01), and a roof prism (Thorlabs, PS951). The fibers will cause random polarization degradation, and the reflection on the metal reflector will add phase delay and basis vector rotation to the polarized beam; Also, the multiple total internal reflections inside the roof prism will add phase delay to the polarized beam. Multiple sets of compensation experimental results are obtained by changing the placement of single-mode fiber to verify method repeatability.

**Fig. 5:**
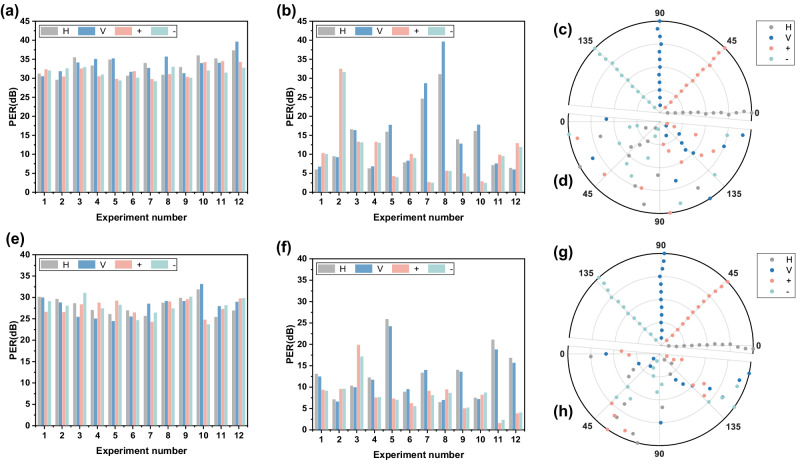
Experimental results. **a** The PER of fiber optical link after compensation; (**b**) The PER of fiber optical link before compensation; (**c**) The polarization angle of fiber optical link after compensation; (**d**) The polarization angle of fiber optical link before compensation; (**e**) The PER of simulated satellite optical link after compensation; (**f**) The PER of simulated satellite optical link before compensation; (**g**) The polarization angle of simulated satellite optical link after compensation; (**h**) The polarization angle of simulated satellite optical link before compensation.

The experimental results are shown in Fig. 5:

For the fiber optical link, the method can achieve compensation for the minimum polarization extinction ratio of 28.4 dB (690:1) and an average of 32.2 dB (1660:1), which is much higher than the 23 dB reported in ref. ^28^. The angles of the four polarization states after compensation are basically consistent with theory, indicating a good compensation effect.

For the simulated satellite optical link, the method can achieve the polarization extinction ratio of at least 23.7 dB (234:1) and an average of 27.9 dB (617:1), which basically meets the polarization maintaining requirements for the satellite terminal as described in reference^11^. The angles of the four polarization states after compensation are consistent with the prediction from theory, indicating good compensation effects. It is worth noting that in the experiment, the introduction of fibers and the difference in reflectivity between p-polarized light (parallel) and s-polarized light (perpendicular) of mirrors will cause depolarization, which will weaken the effect of control methods. In practice, the optical link between controller and detector is mainly composed of mirrors, and appropriate mirror coating designs will improve the compensating effect of this method.

## Conclusions

This paper proposes a real-time polarization compensation method based on channel Muller parameters detection. In this method, the Muller parameters of the optical link are first detected using a single polarization detector, and then the optimal parameters of the polarization controller for achieving control are obtained by applying the gradient descent algorithm. Simulation and experimental results demonstrate numerous advantages:

- High precision. Simulation results indicate an equivalent compensation effect to the prevailing SPGD feedback method and heuristic polarization compensation method, and it can be applied to free space optical link. Furthermore, experimental data confirm its superior compensation efficacy compared to other published results, meeting engineering requirements.
- Faster and able to achieve real-time compensation. Simulation results reveal that this method’s speed is comparable to the heuristic polarization compensation method and faster than the SPGD feedback method. When applied in satellite-ground optical link, the control process can be completed in a very short time, with time consumption considerably lower than satellite pass durations. Notably, the speed of polarization control correlates positively with the number of detections, and we believe that our method has the minimum number of detections among all methods. For achieving control of four polarization states, knowledge of parameters within the Mueller transmission matrix is essential. In our method, the solution of these parameters is transformed into solving a system of linear equations. According to the constraints between these parameters (such as unitary constraints) and the rules for solving linear equations, the minimum number of detections can be obtained.
- Broad applicability and universality. This method boasts a wide array of potential application scenarios since this method is derived based on a universal polarization transmission model. It can be effectively deployed in any context adhering to this transmission model. This universal applicability sets it apart from the heuristic compensation method, which assumes that the matrix between the polarization controller and the detector is an identity matrix. Consequently, our method addresses this defect and can be employed not only for polarization control in fiber optical links but also in the context of free-space optical links. It is worth noting that both the heuristic and our methods require that the transmission matrix of the optical link obeys unitary transformation, or that the depolarization effect of the optical link is not obvious. Feedback methods like SPGD, which employ the minimum QBER as an objective function, do not have this requirement. Despite this, quantum communication requires that the optical link transmission matrix must be unitary (or approximately unitary).
- Minimum polarization controller usage. In this universal model, the polarization controller is also a universal model. We analyzed the minimum number of each type of polarization controller. The number of EPCs used (delay and fast/slow axes both adjustable) is smaller than the heuristic method, but it is almost the same compensation effect and control speed achieved. However, our method and the heuristic method require that the parameters of the polarization controller are known, while feedback methods such as SPGD can be unknown.

The successful implementation of the method is contributed to the design of polarization maintenance in QKD systems based on polarization encoding. It can help reduce the polarization compensation time for both fiber and free-space optical links, make compensation more automated, and facilitate the development of integrated global QKD quantum communication networks.

The compensation effect of our method is limited by the jitter of the Muller parameters of optical links. The method is very suitable for optical links with parameters that are quasi-statically within the control time, such as fiber and satellite optical systems. The compensation effect and robustness will improve once the parameter jitter reduces. In the future, improving compensation effectiveness will depend on advances in technologies such as parameter fluctuation suppression and maintaining good unitarity performance of the transmission matrix.

## Methods

### Polarization control principle based on channel Muller parameters detection

Regardless of link types, a universal polarization optical link model can be represented as follows^33^

5 $${M}_{P}={M}_{2}{M}_{l}{M}_{1}$$

*M*_l_ is the transmission matrix of the polarization controller, *M*_1_ is the transmission matrix of the optical link between source and controller, and *M*_2_ is the transmission matrix between controller and detector. When photons pass through *M*_*P*_, they will hit the detector.

This paper adopts the Mueller–Stokes calculus^33^ to represent the model, which provides a clear and intuitive calculation. The optical link typically consists of components such as fibers, lenses, and mirrors, and their rotational movements. The polarization matrix of the optical links can be regarded as a unitary transformation^9,28^ without depolarization. *M*_1_, *M*_2_, and *M*_*l*_ are represented as follows:

6 $${M}_{1}=\left(\begin{array}{ccc}a & b & c\\ d & e & f\\ g & h & i\end{array}\right),{M}_{2}=\left(\begin{array}{ccc}A & B & C\\ D & E & F\\ G & H & I\end{array}\right),{M}_{l}=\left(\begin{array}{ccc}{l}_{11} & {l}_{12} & {l}_{13}\\ {l}_{21} & {l}_{22} & {l}_{23}\\ {l}_{31} & {l}_{32} & {l}_{33}\end{array}\right)$$

For the BB84 protocol^1^, it is necessary to maintain good polarization states in the four directions of 0° (H), 90°(V), +45° ( + ), and −45° (−). After passing through *M*_*P*_, the 0° linearly polarized light represented by $${{{\boldsymbol{H}}}}={\left[\begin{array}{ccc}1 & 0 & 0\end{array}\right]}^{T}$$ will degrade into:

7 $${H}_{{de}}=\left[\begin{array}{c}{Aa}{l}_{11}+{{Adl}}_{12}+{Ag}{l}_{13}+{Bal}_{21}+{{Bdl}}_{22}+{Bg}{l}_{23}+{Ca}{l}_{31}+{{Cdl}}_{32}+{Cg}{l}_{33}\\ {Dal}_{11}+{{Ddl}}_{12}+{Dg}{l}_{13}+{Eal}_{21}+{{Edl}}_{22}+{Eg}{l}_{23}+{Fa}{l}_{31}+{{Fdl}}_{32}+{Fg}{l}_{33}\\ {Ga}{l}_{11}+{{Gdl}}_{12}+{Gg}{l}_{13}+{Hal}_{21}+{{Hdl}}_{22}+{Hg}{l}_{23}+{Ia}{l}_{31}+{{Idl}}_{32}+{Ig}{l}_{33}\end{array}\right]$$

And the +45° linearly polarized light represented by $${{{\boldsymbol{D}}}}={\left[\begin{array}{ccc}0 & 1 & 0\end{array}\right]}^{T}$$ will degrade into:

8 $${D}_{{de}}=\left[\begin{array}{c}{Ab}{l}_{11}+{{Ael}}_{12}+{Ah}{l}_{13}+{{Bbl}}_{21}+{{Bel}}_{22}+{Bh}{l}_{23}+{Cb}{l}_{31}+{{Cel}}_{32}+{Ch}{l}_{33}\\ {{Dbl}}_{11}+{{Del}}_{12}+{Dh}{l}_{13}+{{Ebl}}_{21}+{{Eel}}_{22}+{Eh}{l}_{23}+{Fb}{l}_{31}+{{Fel}}_{32}+{Fh}{l}_{33}\\ {{Gbl}}_{11}+{{Gel}}_{12}+{Gh}{l}_{13}+{{Hbl}}_{21}+{{Hel}}_{22}+{Hh}{l}_{23}+{Ib}{l}_{31}+{{Iel}}_{32}+{Ih}{l}_{33}\end{array}\right]$$

For polarization control, it requires controlling *M*_*l*_ such that the first row of *H*_*de*_ equals 1 and the second row of *D*_*de*_ equals 1, which means achieving:

9 $$\left\{\begin{array}{c}{Aa}{l}_{11}+{{Adl}}_{12}+{Ag}{l}_{13}+{Bal}_{21}+{{Bdl}}_{22}+{Bg}{l}_{23}+{Ca}{l}_{31}+{{Cdl}}_{32}+{Cg}{l}_{33}=1\\ {{Dbl}}_{11}+{{Del}}_{12}+{Dh}{l}_{13}+{{Ebl}}_{21}+{{Eel}}_{22}+{Eh}{l}_{23}+{Fb}{l}_{31}+{{Fel}}_{32}+{Fh}{l}_{33}=1\end{array}\right.$$

It is expressed as a vector as follows:

10 $$\left\{\begin{array}{c}{{{\boldsymbol{l}}}}{{{{\boldsymbol{p}}}}}_{{{{\boldsymbol{H}}}}}=1\\ {{{\boldsymbol{l}}}}{{{{\boldsymbol{p}}}}}_{{{{\boldsymbol{D}}}}}=1\end{array}\right.$$

Where $${{{\boldsymbol{l}}}}{{{\boldsymbol{=}}}}\left[{l}_{11}{{{\boldsymbol{,}}}}\, {l}_{12}{{{\boldsymbol{,}}}}\, {l}_{13}{{{\boldsymbol{,}}}}{{\ldots }}{{{\boldsymbol{,}}}}{l}_{33}\right]\!{{,}}\, {{{{\boldsymbol{p}}}}}_{{{{\boldsymbol{H}}}}}={\left[{Aa},{Ad},{Ag},\ldots ,{Cg}\right]}^{T},{{{{\boldsymbol{p}}}}}_{{{{\boldsymbol{D}}}}}=[{Db},De,Dh,\ldots ,{Fh}]^{T}$$.

To satisfy Eq. (10), it is necessary to obtain the parameters of ***p***_***H***_ and ***p***_***D***_.

Detectors with four polarization directions will measure the Stokes parameters of *H*_*de*_ and *D*_*de*_. Given the adjustable and known nature of the polarization controller’s parameters, it is possible to create multiple linear equations involving ***p***_***D***_, ***p***_***D***_ and the measured values. This is achieved by adjusting the polarization controller’s parameters multiple times. Solving the system of equations yields the values of ***p***_***H***_ and ***p***_***D***_. The formulated system of equations is represented as follows:

11 $$\left\{\begin{array}{c}C\cdot {{{{\boldsymbol{p}}}}}_{{{{\boldsymbol{H}}}}}={{{{\boldsymbol{M}}}}}_{{{{\boldsymbol{HH}}}}}\\ C\cdot {{{{\boldsymbol{p}}}}}_{{{{\boldsymbol{D}}}}}={{{{\boldsymbol{M}}}}}_{{{{\boldsymbol{DD}}}}}\end{array}\right.$$

${{{{\boldsymbol{M}}}}}_{{{{\boldsymbol{HH}}}}}={[{M}_{{HH}}^{1},{M}_{{HH}}^{2},{M}_{{HH}}^{3},\ldots ,{M}_{{HH}}^{n}]}^{T}$ is a vector composed of n measurements of the first Stokes parameter of *H*_*de*_; ${{{{\boldsymbol{M}}}}}_{{{{\boldsymbol{DD}}}}}={[{M}_{{DD}}^{1},{M}_{{DD}}^{2},{M}_{{DD}}^{3},\ldots ,{M}_{{DD}}^{n}]}^{T}$ is a vector composed of n measurements of the second Stokes parameter of *D*_*de*_. C is the parameter matrix of the polarization controller, which is a known quantity. C is represented as follows:

12 $$C=\left[\begin{array}{c}{{{{\boldsymbol{l}}}}}^{{{{\boldsymbol{1}}}}}\\ {{{{\boldsymbol{l}}}}}^{{{{\boldsymbol{2}}}}}\\ \begin{array}{c}\ldots \\ {{{{\boldsymbol{l}}}}}^{{{{\boldsymbol{n}}}}}\end{array}\end{array}\right]=\left(\begin{array}{ccc}{l}_{11}^{1} & {l}_{12}^{1} & \begin{array}{cc}\ldots & {l}_{33}^{1}\end{array}\\ \begin{array}{c}{l}_{11}^{2}\\ \ldots \end{array} & \begin{array}{c}{l}_{12}^{2}\\ \ldots \end{array} & \begin{array}{cc}\begin{array}{c}\ldots \\ \ldots \end{array} & \begin{array}{c}{l}_{33}^{2}\\ \ldots \end{array}\end{array}\\ {l}_{11}^{n} & {l}_{12}^{n} & \begin{array}{cc}\ldots & {l}_{33}^{n}\end{array}\end{array}\right)$$

After the polarized light in the H direction passes through MP with the polarization controller parameters set at ***l*** ^***i***^, the photon counts on the detectors in the H and V directions are denoted as ${T}_{{HH}}^{i}$ and ${T}_{{HV}}^{i}$, respectively. Similarly, when the polarized light in the + direction goes through MP under the same controller settings, the photon counts on the detectors in the + and − directions are denoted as ${T}_{{DD}}^{i}$ and${T}_{{DA}}^{i}$. According to the Mueller-Stokes calculus^33^, the relationships between the values of ${M}_{{HH}}^{i}$, ${M}_{{DD}}^{i}$ and the photon counts are expressed as^33^

13 $$\begin{array}{c}{M}_{{HH}}^{i}=\frac{{T}_{{HH}}^{i}-{T}_{{HV}}^{i}}{{T}_{{HH}}^{i}+{T}_{{HV}}^{i}},1\le i\le n\\ {M}_{{DD}}^{i}=\frac{{T}_{{DD}}^{i}-{T}_{{DA}}^{i}}{{T}_{{DD}}^{i}+{T}_{{DA}}^{i}},1\le i\le n\end{array}$$

If the parameters of both *M*_1_ and *M*_2_ are unknown, *n* ≥ 9. When either *M*_1_ or *M*_2_ is an identity matrix, *n* ≥ 3.

Note that since the matrices mentioned all conform to unitary transformations, there exists:

14 $$\left\{\begin{array}{c}\begin{array}{c}{a}^{2}+{d}^{2}+{g}^{2}=1\\ {A}^{2}+{B}^{2}+{c}^{2}=1\\ {D}^{2}+{E}^{2}+{F}^{2}=1\end{array}\\ {b}^{2}+{e}^{2}+{h}^{2}=1\end{array}\right.$$

Upon introducing this constraint, both the minimum value of n and the number of measurements decrease. In scenarios where *M*_2_ is the unit matrix, typical in the context of most fiber-optical links, *n* ≥ 2. When H and D polarized light pass through the *M*_*P*_, the detectors in the four polarization directions will measure the first two Stokes parameters of *H*_*de*_ and the first two Stokes parameters of *D*_*de*_ at least twice respectively. Subsequently, the values of ***p***_***H***_ and ***p***_***D***_ will be computed.

If the parameters of both *M*_1_ and *M*_2_ are unknown, the number of measurements will also be reduced. After ***p***_***H***_ is solved, the absolute values of [*A, B, C, a, d, g*] can be obtained through Eq. (14), although their corresponding positive or negative signs cannot be determined. When the polarized light output by the light source is H, the absolute values of the second-row parameters [*D, E, F, a, d, g*] of *D*_*de*_ are simultaneously determined based on the photon count in both the + and − directions. Consequently, this result is combined with ***p***_***H***_, leading to the determination of the values of [*A, B, C, D, E, F, a, d, g*]. To solve for [*b, e, h*] in ***p***_***D***_, at least second measurements are required.

The transformation represented by the Mueller matrix can be visualized as a rotation of the coordinate axis in the Poincaré sphere. The orthogonal relationship between the *R*-axis, *H*-axis, and *D*-axis remains intact after undergoing the unitary transformation. Therefore, there exists:

15 $$\left\{\begin{array}{c}{M}_{1}{{{\boldsymbol{H}}}}\times {M}_{1}{{{\boldsymbol{D}}}}={M}_{1}{{{\boldsymbol{R}}}}\\ {M}_{2}{{{\boldsymbol{H}}}}\times {M}_{2}{{{\boldsymbol{D}}}}={M}_{2}{{{\boldsymbol{R}}}}\end{array}\right.$$

$${{{{\boldsymbol{R}}}}=\left[\begin{array}{ccc}0 & 0 & 1\end{array}\right]}^{T}$$ is right-circularly polarized light. According to Eq. (15), it is easy to calculate the values of [*G, H, I*] and [*c, f, i*]. Therefore, all parameters in *M*_1_ and *M*_2_ can be determined.

Once the channel parameters have been measured, the optimal parameters can be obtained by solving Eq. (9). To minimize compensation errors, the optimization objective also considers the polarization R. The optimal parameter of controller ***l*** ^*^ can be determined as follows:

16 $${{{{\boldsymbol{l}}}}}^{{{{\boldsymbol{* }}}}}=\mathop{{argmin}}\limits_{{{{\boldsymbol{l}}}}}\left[{\left({{{\boldsymbol{l}}}}{{{{\boldsymbol{p}}}}}_{{{{\boldsymbol{H}}}}}-1\right)}^{2}+{\left({{{\boldsymbol{l}}}}{{{{\boldsymbol{p}}}}}_{{{{\boldsymbol{D}}}}}-1\right)}^{2}+{({{{\boldsymbol{l}}}}{{{{\boldsymbol{p}}}}}_{{{{\boldsymbol{R}}}}}-1)}^{2}\right]$$

**Fig. 6:**
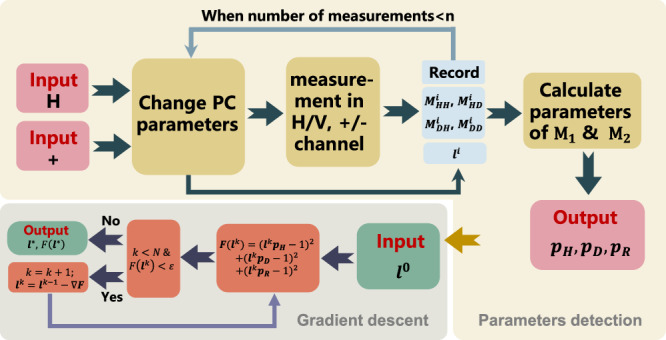
Polarization control method based on channel Muller parameters detection. Flow diagram of the method.

***p***_***D***_ is the parameter array in the second row of *D*_*de*_, ***p***_***R***_ is the parameter array in the second row of *R*_*de*_. It is easy to solve for the value of ***l*** ^*^ using optimization algorithms such as gradient descent. Our method can be summarized in the Fig. 6.

The parameter detection method can also be applied to the polarization compensation method for the waveplate group described in refs. ^9,28^, which requires knowledge of the exact values of Mueller matrices for each system to obtain an analytical solution for the compensation angle.

### Scheme for polarization controller selection

The simplification of waveplate combinations in various types of polarization controllers will be considered. The degradation of polarization can be characterized as the rotation of Stokes parameter coordinate axes in Poincaré sphere. The function of *M*_*l*_ is to restore these degraded axes to their original position. If compensation is implemented, Eq. (5) will be expressed as

17 $${M}_{2}{M}_{l}{M}_{1}=E$$

*E* is a 3 × 3 identity matrix. The solution of this model is equal to the following equation:

18 $${M}_{l}{M}_{1}{M}_{2}=E$$

Thus, the sufficient and necessary condition for restoring the axis in Eq. (13) is to restore the axis rotation caused by *M*_1_ *M*_2_.

A single waveplate contains only two degrees of freedom. Although these two variables can restore a certain axis in the Poincaré sphere to the original position, the positions of other axes are also determined at this time. Thus, the third degree of freedom needs to be introduced.

For the waveplate with adjustable delay and rotatable fast/slow axes, at least two waveplates can reset the rotation of the three coordinate axes caused by *M*_1_ *M*_2_, such as ${M}_{c}={M}_{0,{\delta }_{2}}{M}_{{\theta }_{1},{\delta }_{1}}$. The waveplate ${M}_{{\theta }_{1},{\delta }_{1}}$ is used to reset the H axis, and the waveplate ${M}_{0,{\delta }_{2}}$ is used to reset the H and R axes. The Mueller matrix of the waveplate is expressed as:

19 $${M}_{{\theta }_{i},{\delta }_{i}}=\left(\begin{array}{ccc}{\cos }^{2}2{\theta }_{i}+{\sin }^{2}2{\theta }_{i}\cos {\delta }_{i} & \cos 2{\theta }_{i}\sin 2{\theta }_{i}\left[1-\cos {\delta }_{i}\right] & \sin 2{\theta }_{i}\sin {\delta }_{i}\\ \cos 2{\theta }_{i}\sin 2{\theta }_{i}\left[1-\cos {\delta }_{i}\right] & {\cos }^{2}2{\theta }_{i}\cos {\delta }_{i}+{\sin }^{2}2{\theta }_{i} & -\cos 2{\theta }_{i}\sin {\delta }_{i}\\ -\sin 2{\theta }_{i}\sin {\delta }_{i} & \cos 2{\theta }_{i}\sin {\delta }_{i} & \cos {\delta }_{i}\end{array}\right)$$

For waveplates with a single variable parameter that has adjustable delay or a rotatable fast/slow axis, three waveplates are at least required to provide the three degrees of freedom. There are many cases with three waveplates about the fast/slow axis rotatable waveplate group9, such as ${M}_{c}={M}_{{\theta }_{3},\pi }{M}_{{\theta }_{2},\frac{\pi }{2}}{M}_{{\theta }_{1},\frac{\pi }{2}}$. For the delay adjustable waveplate group, such as ${M}_{c}={M}_{0,{\delta }_{3}}{M}_{\frac{\pi }{4},{\delta }_{2}}{M}_{0,{\delta }_{1}}$, where the function of ${M}_{0,{\delta }_{1}}$ is to make the second parameter of *H* axis equal to 0, the function of ${M}_{\frac{\pi }{4},{\delta }_{2}}$ is to reset *H* axis, and the function of ${M}_{0,{\delta }_{3}}$ is to reset D and R axes. A summary is shown in Table 3:

**Table 3:** Comparison of polarization controllers

Type	Typical device	Minimum number of components	Application optical link
Delay adjustable	EPC, LCVR	3	Free-space, Fiber
Fast/Slow axes rotatable	Crystal WPG, EPC	3	Satellite, Free-space, Fiber
Delay adjustable & Fast/Slow axes rotatable	EPC	2	Fiber

## Data Availability

Raw experimental data and calculations can be obtained from the corresponding author upon a reasonable request.

## Code availability

All relevant computing codes that support the findings of this study are available from the corresponding authors upon request.
